# Distribution and Environmental Drivers of Fungal Denitrifiers in Global Soils

**DOI:** 10.1128/spectrum.00061-23

**Published:** 2023-05-24

**Authors:** Yvonne Bösch, Grace Pold, Aurélien Saghaï, Magnus Karlsson, Christopher M. Jones, Sara Hallin

**Affiliations:** a Swedish University of Agricultural Sciences, Department of Forest Mycology and Plant Pathology, Uppsala, Sweden; State Key Laboratory of Mycology, Institute of Microbiology, Chinese Academy of Sciences

**Keywords:** biogeography, denitrification, *nirK*, nitrous oxide, pathogenic fungi, phyloecology, terrestrial fungi

## Abstract

The microbial process of denitrification is the primary source of the greenhouse gas nitrous oxide (N_2_O) from terrestrial ecosystems. Fungal denitrifiers, unlike many bacteria, lack the N_2_O reductase, and thereby are sources of N_2_O. Still, their diversity, global distribution, and environmental determinants, as well as their relative importance, compared to bacterial and archaeal denitrifiers, remain unresolved. Employing a phylogenetically informed approach to analyze 1,980 global soil and rhizosphere metagenomes for the denitrification marker gene *nirK*, which codes for the copper dependent nitrite reductase in denitrification, we show that fungal denitrifiers are sparse, yet cosmopolitan and that they are dominated by saprotrophs and pathogens. Few showed biome-specific distribution patterns, although members of the Fusarium oxysporum species complex, which are known to produce substantial amounts of N_2_O, were proportionally more abundant and diverse in the rhizosphere than in other biomes. Fungal denitrifiers were most frequently detected in croplands, but they were most abundant in forest soils when normalized to metagenome size. Nevertheless, the overwhelming dominance of bacterial and archaeal denitrifiers suggests a much lower fungal contribution to N_2_O emissions than was previously estimated. In relative terms, they could play a role in soils that are characterized by a high carbon to nitrogen ratio and a low pH, especially in the tundra as well as in boreal and temperate coniferous forests. Because global warming predicts the proliferation of fungal pathogens, the prevalence of potential plant pathogens among fungal denitrifiers and the cosmopolitan distribution of these organisms suggest that fungal denitrifier abundance may increase in terrestrial ecosystems.

**IMPORTANCE** Fungal denitrifiers, in contrast to their bacterial counterparts, are a poorly studied functional group within the nitrogen cycle, even though they produce the greenhouse gas N_2_O. To curb soil N_2_O emissions, a better understanding of their ecology and distribution in soils from different ecosystems is needed. Here, we probed a massive amount of DNA sequences and corresponding soil data from a large number of samples that represented the major soil environments for a broad understanding of fungal denitrifier diversity at the global scale. We show that fungal denitrifiers are predominantly cosmopolitan saprotrophs and opportunistic pathogens. Fungal denitrifiers constituted, on average, 1% of the total denitrifier community. This suggests that earlier estimations of fungal denitrifier abundance, and, thereby, it is also likely that the contributions of fungal denitrifiers to N_2_O emissions have been overestimated. Nevertheless, with many fungal denitrifiers being plant pathogens, they could become increasingly relevant, as soilborne pathogenic fungi are predicted to increase with ongoing climate change.

## INTRODUCTION

Terrestrial ecosystems are major sources of the long-lived, stratospheric, ozone-depleting substance and greenhouse gas nitrous oxide (N_2_O). Direct emissions from natural soils contribute 58% of the total natural fluxes of N_2_O to the atmosphere (a total of 9.7 Tg N year^−1^), while agricultural soils account for 32% of the anthropogenic sources (a total of 7.3 Tg N year^−1^), with global emissions currently increasing by 2% per decade (1). Nitrous oxide primarily originates from the microbial process denitrification (2, 3). This process is an alternative to aerobic respiration when oxygen levels are low, and reduces nitrate to di-nitrogen (N_2_) in four consecutive reactions. It has predominantly been studied in bacteria, but some archaea and fungi are also known as denitrifiers (4). Denitrifying fungi are of particular interest, as to date, no species has been reported to encode the N_2_O reductase that catalyzes the reduction of N_2_O to N_2._ Therefore, fungi terminate denitrification with N_2_O and are potentially important sources of N_2_O from soil. Most fungi for which N_2_O production has been demonstrated belong to the fungal classes Eurotiomycetes and Sodariomycetes, including the genera Fusarium, Aspergillus, Bionectria, and Trichoderma, of which many are putative pathogens (5, 6). However, our knowledge about their ecology and distribution in terrestrial biomes is limited, which underpins their capacity to contribute to N_2_O emissions and limits our understanding of their role in terrestrial nitrogen (N) feedback to the climate system.

Fungal denitrification has been reported in a range of different terrestrial ecosystems (7–12), although its contribution to total denitrification and N_2_O production varies across these systems (13). In a few cases, fungal denitrification has been suggested to be more prevalent than bacterial denitrification (e.g., in grasslands), and it may increase in agricultural soils, depending on management practices (7, 8, 14). Although increased carbon source complexity (15), lower soil pH (13, 16–19), and low oxygen levels have been shown to favor fungal denitrification (18, 20), broadly conserved edaphic factors that select for fungal or prokaryotic denitrifiers, if any, have not yet been established, as current knowledge is based on a limited number of case studies. Therefore, the extant diversity of fungal denitrifiers as well as the terrestrial habitats in which fungal denitrifiers thrive, remain uncertain.

Here, we present a comprehensive, phylogenetically informed analysis of terrestrial fungal denitrifiers on a global scale. We examine their abundance and the distribution of fungal denitrifier genotypes in 1,980 soil and rhizosphere metagenomes that represent 608 sampling sites of the major terrestrial biomes (Table S1). Utilizing the *nirK* gene, which encodes the copper-dependent nitrite reductase that is involved in denitrification, as a marker gene for fungal denitrifiers, we circumvent the current debate about the involvement of the fungal nitric oxide reductase P450*nor* in other functions (e.g., secondary metabolism) (21). The abundance of fungal *nirK* was assessed both per total number of reads and in relation to the overall fungal community, based on the fungal 18S rRNA gene counts, to determine biome-specific differences, the edaphic drivers of fungal *nirK* counts, and the proportion of fungal denitrifiers within the overall fungal community. Further, we compared differences across biomes and evaluated the edaphic drivers of the fungal, relative to bacterial and archaeal, denitrifiers as a measure of their relative capacity for denitrification. The compilation and the use of a large, globally distributed data set of metagenomes and associated metadata allowed us to address the ecology, biogeography, and abundance of fungal denitrifiers across broad environmental gradients and free from PCR-introduced biases. Further, the comprehensive *nirK* reference phylogeny that was used to recruit *nirK* genes from metagenomes provides a phylogenetic framework for research on the ecology and evolution of denitrification.

## RESULTS

### Biome-related patterns of fungal denitrifier abundance.

**FIG 1:**
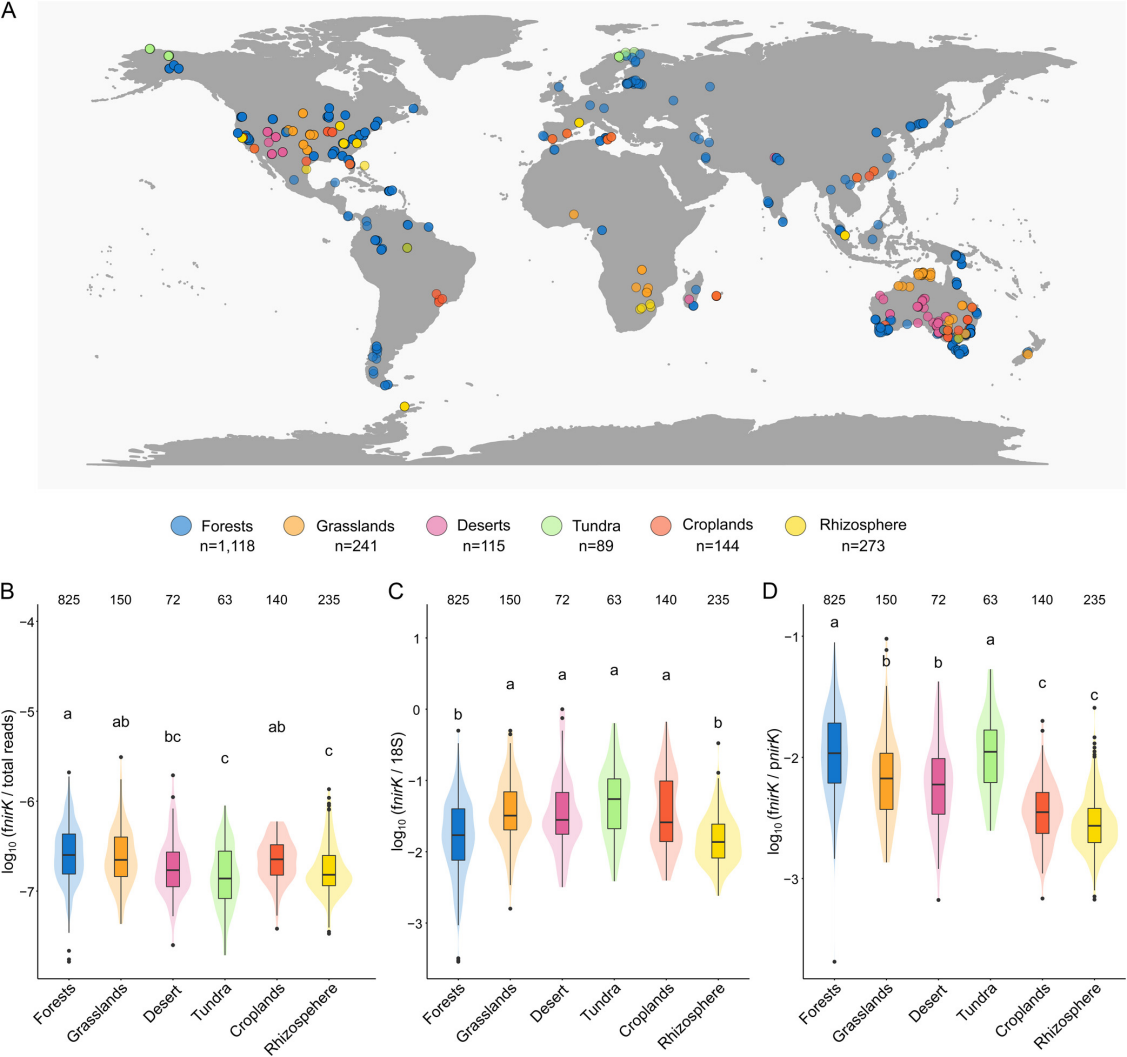
Origins of metagenomes and abundance of fungal *nirK* in terrestrial biomes. (A) 1,980 metagenomes representing 608 sampling locations across the globe. The sampling locations of 41 rhizosphere samples are not indicated due to the absence of associated geographic coordinates. Made with Natural Earth. (B) Fungal *nirK* (f*nirK*) gene fragment counts, normalized to the total number of reads per metagenome. (C) Abundance of f*nirK*, relative to fungal 18S rRNA gene (18S) fragment counts. (D) Abundance of fungal, relative to prokaryotic, *nirK* (p*nirK*) gene fragment counts. Different letters indicate significant differences between biomes (ANOVA, Šidák-corrected pairwise comparisons, *P* < 0.05). The numbers above the boxplots in panels B to D indicate the number of metagenomes within each biome with at least one fungal *nirK* hit. The box limits represent the interquartile range (IQR), with the median values being represented by the centerline. Whiskers represent values that are ≤1.5 times the upper and lower quartiles, whereas points indicate values outside this range. The shaded areas show kernel density estimations, indicating the distribution of the data.

**FIG 2:**
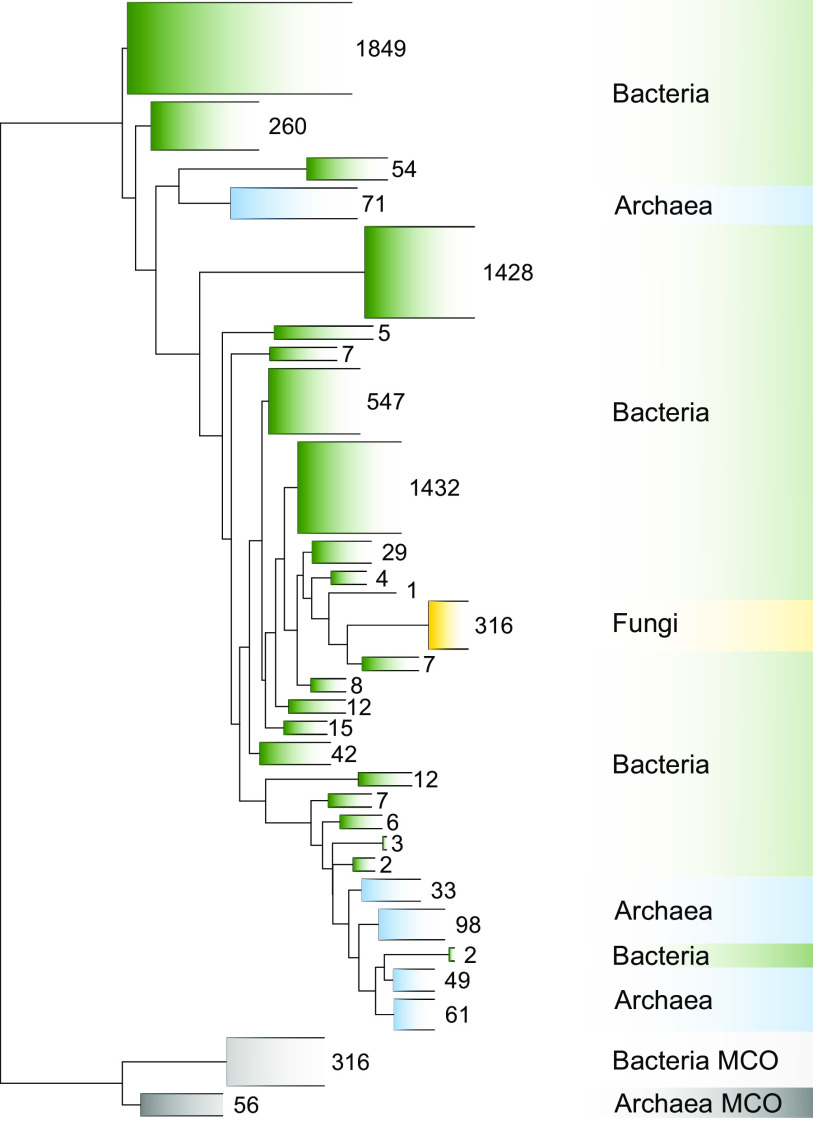
Reference phylogeny of prokaryotic and fungal *nirK*, created from 6,732 full-length genomic *nirK* gene sequences. The phylogeny was determined based on a maximum likelihood analysis of the amino acid sequences of *nirK*, using the LG+G substitution model. The phylogeny was subsequently used as the reference tree for the phylogenetic placement of *nirK* gene fragments that were retrieved from 1,980 metagenomes. Clades were collapsed, and the number of species per collapsed clade is indicated. The outgroups consist of multicopper oxidase (MCO) sequences originating from cyanobacterial and thaumarcheotal genomes.

To investigate the prevalence of fungal denitrifiers in terrestrial ecosystems, a collection of 1,980 metagenomes derived from the tundra, forests, grasslands and savannas, deserts, croplands, and rhizosphere of 14 plant taxa was analyzed (Fig. 1A; Table S1). Based on a probabilistic approach for the phylogenetic placement of fragments of the fungal and prokaryotic denitrifier marker gene *nirK* in the metagenomes into a *nirK* reference tree with 6,732 sequences (Fig. 2), archaeal, bacterial, and fungal *nirK* fragments were detected in 97, 100, and 76% of the metagenomes, respectively. Fungal *nirK* accounted for 4.5 ± 6.9 (mean ± standard deviation [SD]) gene fragment counts per metagenome, assigning the value “0” when fungal *nirK* was not detected. The fungal *nirK* counts were nearly 10 and 200 times lower than those for archaea and bacteria, respectively. In contrast, fungal 18S rRNA gene fragments were identified in 99% of the metagenomes, with an average of 457 ± 1,097 reads per metagenome. The detection of fungal *nirK* differed among biomes, with the lowest proportion of metagenomes with zero fungal *nirK* fragments being found in cropland metagenomes (2%) and the highest being found in deserts (37%) (Fig. S1A). These zero-counts may not indicate the absence of fungal *nirK* but could potentially be the result of undersampling, as suggested by the lower numbers of total reads that were consistently recorded for biome-specific metagenomes in which fungal *nirK* was not detected (Fig. S1B). Hence, for the subsequent analyses, we retained the 1,485 metagenomes containing both fungal *nirK* and fungal 18S rRNA gene fragment counts, which correspond to 825 forest, 150 grassland, 140 cropland, 72 desert, 63 tundra, and 235 rhizosphere metagenomes. In this subset, fungal *nirK* accounted for 6.0 ± 7.4 (mean ± SD) gene fragment counts per metagenome.

When the metagenomes were grouped according to their biome classifications at Level 2 (Table S1), the total abundance of fungal *nirK*, normalized to the total number of reads so as to account for the variation in the sequencing depth, was highest in forests and lowest in the tundra and rhizosphere (Fig. 1B). Grouping at a refined biome classification (Level 3) showed that the high fungal *nirK* abundance in forests was driven by the abundance in tropical and subtropical moist broadleaf forests (Table 1). The fungal *nirK* abundance was significantly higher in these biomes, compared with Mediterranean forests, woodlands, and shrublands, which were similar to deserts, the tundra, and the rhizosphere. In contrast, the proportion of fungi carrying *nirK*, which was defined as the ratio of fungal *nirK* counts to those of the fungal 18S rRNA gene, was lower in forests and in the rhizosphere, compared to other biomes (Fig. 1C). Comparisons at biome Level 3 revealed that the gene ratios were the highest in tropical and subtropical dry broadleaf forests and were the lowest in Mediterranean forests and shrublands (Table 1).

**TABLE 1:** Abundance of fungal *nirK* (f*nirK*) and fungal 18S rRNA genes (18S) in terrestrial biomes at Level 3^*a*^

Biomes at Level 3 (*n*)	Fungal *nirK* (10^−7^)	Fungal 18S (10^−7^)	f*nirK*:18S (%)	f*nirK*:p*nirK* (%)
Boreal forests and taiga (59)	3.5 ± 2.9 ^bcd^	412 ± 296 ^b^	1.6 ± 2.1 ^f^	1.7 ± 1.4 ^b^
Temperate coniferous forests (224)	3.4 ± 2.9 ^bc^	282 ± 364 ^c^	2.7 ± 2.8 ^de^	1.5 ± 1.3 ^b^
Temperate broadleaf and mixed forests (417)	3.5 ± 2.9 ^bc^	227 ± 217 ^cd^	3.2 ± 4.4 ^d^	1.5 ± 1.3 ^bc^
Mediterranean forests, woodlands, and shrublands (60)	2.1 ± 1.1 ^def^	269 ± 234 ^bcd^	1.4 ± 1.4 ^ef^	1.0 ± 0.7 ^cd^
Tropical and subtropical dry broadleaf forests (28)	2.9 ± 1.6 ^bcde^	87 ± 107 ^ef^	0.8 ± 12.8 ^b^	0.6 ± 0.4 ^cd^
Tropical and subtropical moist broadleaf forests (37)	4.7 ± 3.8 ^b^	194 ± 193 ^cd^	3.9 ± 4.0 ^bcd^	1.6 ± 1.2 ^bc^
Temperate grasslands, savannas, and shrublands (116)	3.3 ± 3.6 ^bcd^	78 ± 76 ^e^	7.2 ± 9.7 ^bc^	0.9 ± 1.2 ^d^
Tropical and subtropical grasslands, savannas, and shrublands (34)	3.0 ± 3.2 ^bcdef^	153 ± 198 ^cde^	4.3 ± 6.6 ^cd^	1.2 ± 1.4 ^bcd^
Deserts and xeric shrublands (72)	2.6 ± 2.8 ^def^	82 ± 89 ^e^	9.2 ± 19.6 ^bc^	0.8 ± 0.7 ^d^
Tundra (63)	1.9 ± 1.6 ^f^	45 ± 46 ^f^	9.2 ± 11.7 ^b^	1.4 ± 1.1 ^bc^
Croplands (140)	2.5 ± 1.3 ^cde^	115 ± 126 ^e^	7.3 ± 10.1 ^bc^	0.4 ± 0.3 ^e^
Rhizosphere (235)	2.2 ± 2.0 ^ef^	146 ± 104 ^d^	2.1 ± 2.8 ^de^	0.3 ± 0.3 ^e^

a The fungal *nirK* fragment counts were normalized by the total number of reads in the corresponding metagenome and were also standardized to the fungal 18S rRNA gene abundance (f*nirK*:18S) as well as that of the prokaryotic *nirK* (p*nirK;* f*nirK*:p*nirK*) (mean ± SD, *n* = 28 to 417). The number of metagenomes is shown in parentheses for each biome. The superscript letters indicate significant differences across biomes (*P* < 0.05).

### Identity of fungal denitrifiers across biomes.

**FIG 3:**
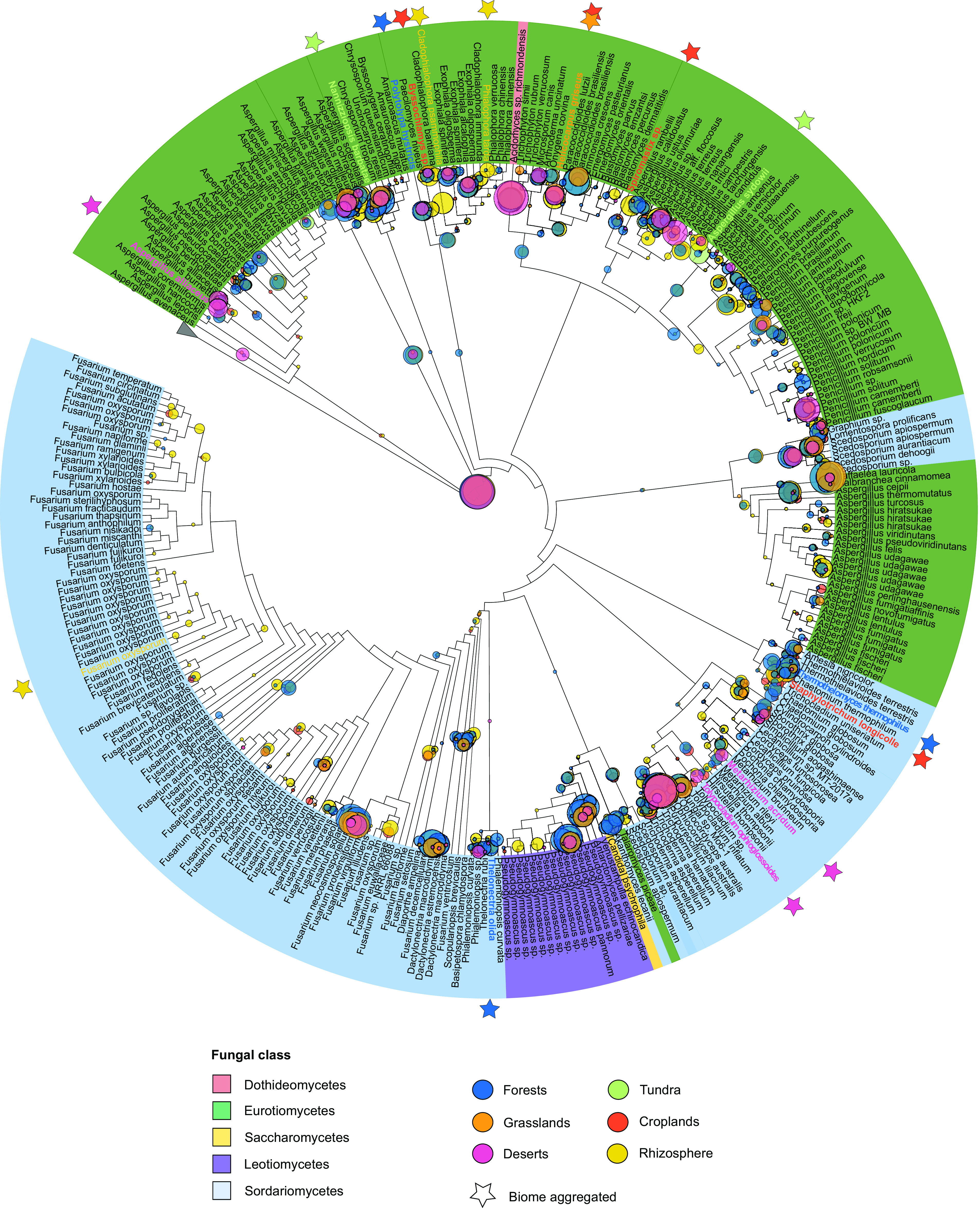
Phylogenetic placements of fungal *nirK* gene fragments detected in soil and rhizosphere biomes within the *nirK* reference cladogram tree. The leaf color indicates the fungal class, and the outgroup sequences are collapsed. The most likely phylogenetic placement for each read is represented by a circle and is colored according to the biome classification at Level 2. The circle size indicates the number of placements on a given tree edge. Stars mark the branches that are enriched in fungal *nirK* placements within a biome, compared to other biomes. Biome-specific placements at Level 2 are shown in Fig. S2.

The fungal *nirK* fragments that were collected from all of the terrestrial biomes spanned the reference tree, which includes *nirK* that was derived from 316 unique fungal sequences within 5 Ascomycota classes: Eurotiomycetes, Dothideomycetes, Leotiomycetes, Saccharomycetes, and Sordariomycetes (Fig. 3; Fig. S2). The two largest classes, namely, Eurotiomycetes and Sordariomycetes, were dominated by *nirK* sequences that were similar to those in the genera Aspergillus, Fusarium, and Penicillium. With two exceptions, the Fusarium *nirK* sequences formed a monophyletic clade, whereas the Aspergillus *nirK* sequences were split into several clades across the tree and were interleaved by clusters of Eurotiomycetes members, such as *Trichophyton*, *Paracoccidioides*, and *Blastomyces.* A large fraction of the metagenome *nirK* sequences was best placed in the region of the tree corresponding to the *nirK* from species within the Eurotiomycetes. These include regions of the reference tree that corresponded to Aspergillus westerdijkiae (2% of placements), Chrysosporium tropicum (1.7%), Paracoccidioides brasiliensis (1.4%), and several species of the genus Exophiala (1.6%). In addition, placements aligning to the single representative of the class Dothideomycetes, namely, Acidomyces richmondensis (2.4%), were abundant. We also noted high *nirK* counts corresponding to *Antarctomycetes* (1.7%) and *Pseudogymnoascus* (4.9%) within the class Leotiomycetes. Among the Sordariomycetes, the *nirK* fragments that were detected in all terrestrial biomes were frequently placed close to species of Fusarium (particularly F. neocosmosporiellum [3.4%]), *Dactylonectria* (2.5%), and *Scedosporium* (2.5%), as well as Trichoderma hamatum (0.9%), Raffaelea lauricola (4.5%), and Purpureocillium lilacinum (8.1%). A fraction of *nirK* sequences (18.6%) was placed at the basal part of the fungal tree.

A visual comparison of the placements across biomes grouped at Level 2 showed that the forest metagenomes had more pronounced aggregations of placements within the genera Polytolypa, Thermothelomyces, and Thelonectria, which summed up to 0.7%, 1.5% and 0.6% of the placements within forest biomes, respectively (Fig. 3; Fig. S2). Placements of *Helicocarpus* (1.9%) were enriched in grasslands, whereas the desert metagenomes were enriched in *nirK* from the mold Aspergillus alliaceus (2.2%) as well as the genera Tolypocladium (1.1%), and Metarhizium (1.1%). In the tundra metagenomes, placements of *Nannizziopsis* (2.8%) and Aspergillus sydowii (3.3%) were found in comparably high abundance. In croplands, placements within the genera Byssochlamys (0.6%), Helicocarpus (1.4%), Spiromastix (0.4%), and Staphylotrichum (2.2%) were more prominent. In the rhizospheres, the genera Cladophialophora (2.1%) and Phialophora (0.5%) were relatively abundant, compared to other biomes. Furthermore, placements assigned to Fusarium oxysporium were almost exclusively found in rhizosphere metagenomes (1%), whereas placements in this part of the tree were rare for fungal *nirK* that was detected in other biomes (Fig. 3; Fig. S3).

### Environmental drivers of fungal *nirK* abundances.

**FIG 4:**
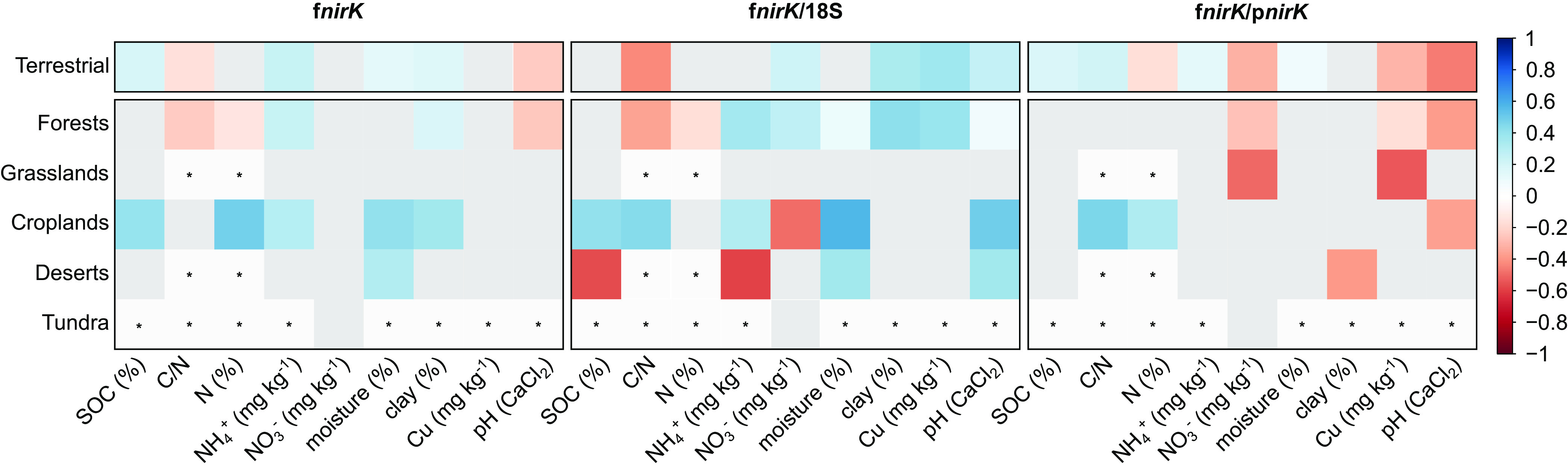
Spearman ranked correlations of fungal *nirK* abundance as well as the ratio to 18S rRNA gene abundance (f*nirK*:18S) and prokaryotic *nirK* (f*nirK*: p*nirK*) with edaphic variables. Correlations are shown for all terrestrial biomes and for five terrestrial biomes at Level 2. Rhizosphere metagenomes were not included due to the absence of associated metadata. The correlations are colored if significant (*P* < 0.05), according to their strength, with red for negative correlations and blue for positive correlations. Nonsignificant correlations are colored in gray. Those not determined due to an insufficient sample number (*n* < 25) are marked with an asterisk. Sample sizes differed among metagenomes, as indicated in Table S2. SOC, soil organic carbon; C:N, total carbon to total nitrogen ratio; N, total nitrogen; NH_4_^+^, ammonium; NO_3_^−^, nitrate; moisture, soil moisture; clay, soil clay content; Cu, soil copper content; pH, soil pH measured in CaCl_2_. NO_3_^−^ in tundra soils was reported in μM.

To determine the environmental factors driving the prevalence of fungal denitrifiers, we related fungal *nirK* counts to metagenome-associated edaphic variables. Across all terrestrial biomes combined, fungal *nirK* abundance was positively correlated with soil organic carbon (SOC), ammonium content, soil moisture, and clay content but was negatively associated with the carbon to nitrogen ratio (C/N) and pH (Fig. 4). The overall associations with C/N and pH were driven by significant correlations with the fungal *nirK* in the forest biomes, and those with SOC were driven by significant correlations with the fungal *nirK* in croplands. Across the biomes classified at Level 2, insignificant or contrasting relationships were observed for some soil variables, but the soil ammonium content, soil moisture, and clay content correlated positively with fungal *nirK* across several biomes. For the soil N content, biome-specific relationships with fungal *nirK* were detected with positive correlations in croplands and negative correlations in forests.

The fraction of the fungal community carrying *nirK* across all biomes was largely related to the same soil factors as was the fungal *nirK* abundance, apart from pH, which showed a positive relationship with the proportion of fungal *nirK* (Table 1). The relationship with pH was consistent for croplands, deserts, and to a lesser extent, forest soils. We also noted that the fraction of fungal *nirK* increased with increasing copper content, which was mainly driven by the forest soils. Among biomes, we detected contrasting associations with the proportion of fungal *nirK* in the fungal community and with SOC, C/N, ammonium, and nitrate (NO_3_^−^). The overall decrease of the proportion of fungal *nirK*, in relation to the C/N ratio, was also observed in forests, whereas croplands showed a positive relationship. Ammonium was significantly correlated with the proportion of fungal *nirK* in both forest and cropland biomes, yet it was negatively correlated in the desert soils. Opposing patterns were also observed for NO_3_^−^ in forests and croplands, with negative associations being found in the latter. For the rhizosphere metagenomes, for which only metadata on the host plant species was available, we noted a significantly lower proportion of fungal *nirK* within the fungal community in the rhizosphere of the Poaceae species, compared to the average of all rhizosphere metagenomes (Fig. S3A).

### Fungal to prokaryotic *nirK* ratio.

The dominance of prokaryotic *nirK* (i.e., the sum of bacterial and archaeal *nirK* fragment sequence counts) was observed across all biomes (Fig. 1D). The highest ratio between fungal and prokaryotic *nirK* was found in forest and tundra soils. Intermediate ratios were detected in grasslands and deserts. The lowest were found in croplands and in the rhizosphere. Within forests, this ratio was significantly higher in boreal forests, the taiga, and temperate coniferous forests (Table 1). For the rhizosphere, the ratio between fungal and prokaryotic *nirK* was significantly lower in the metagenomes from *Miscanthus* sp. and *Populus* sp., compared to the average of all rhizosphere samples (Fig. S3B).

## DISCUSSION

Here, we evaluated the distribution and abundance of fungal *nirK* in 1,980 metagenomes, thereby providing a global survey of fungal denitrifiers in terrestrial ecosystems. Although fungal denitrifiers were rare, overall, compared to their archaeal and bacterial counterparts, they showed biome-specific differences in both abundance and species distributions. Our study further highlights that the dominant soil fungal denitrifiers are cosmopolitan organisms (i.e., found in most soils across the globe) and are adapted to a broad range of climatic and environmental conditions. Known cosmopolitan fungal species carrying *nirK*, such as Aspergillus westerdijkiae, A. sydowii, Penicillium solitum, or Fusarium neocosmosporiellum, were identified in all of the Level 2 biomes. Similarly, Dothideomycetes *nirK* sequences were detected across biomes, despite being represented by a single species (Acidomyces richmondensis) in the *nirK* reference phylogeny. Most members of the Dothideomycetes exhibit a saprotrophic lifestyle, but some are known as pathogens and endophytes (22). Aspergillus and *Penicillium* species have been isolated from a range of environments (23, 24), and they are known for their efficient dispersal strategies (25) and stress tolerance (26, 27). Members of both genera also have the capacity to produce powerful extracellular enzymes for lignin and xylose degradation (28), which is an important trait that supports growth in environments with poor availability of easily accessible C substrates. In contrast to Aspergillus, the distribution of certain *nirK*-carrying Fusarium appears to be more biome specific, as members of the Fusarium oxysporum species complex were proportionally more abundant and diverse in the rhizosphere than in the other biomes. An increased fungal contribution to N_2_O production by denitrification has been assigned to rhizosphere processes (29), and, in particular, F. oxysporum is known to produce substantial amounts of N_2_O (5, 30). F. oxysporum is also a typical root pathogen, suggesting that host-pathogen interactions might play a role for increased fungal denitrifier abundance. Accordingly, there are indications that the fungal nitric oxide reductase P450nor is also involved in fungal virulence (31). Further evidence for the relevance of host-pathogen interactions as a driver of fungal denitrifiers is the relatively high proportion of pathogenic fungi in the reference phylogeny, and our observation was that some of these pathogenic fungi were more associated with certain biomes, based on the placements of the metagenome sequences. This includes the genera *Tolypocladium* and Metarhizium, both of which are known entomopathogens in warm deserts, the plant pathogen species *Byssochlamys*, and *Thelonectria*, which infests hardwoods in forests. Overall, these findings suggest that plant-pathogen interactions support fungal denitrifiers, and a possible mechanism is that denitrification increases virulence, as is shown for several bacteria (32).

The majority of the placements were located close to the leaves of the reference tree, suggesting that the majority of the metagenome-derived fungal denitrifiers are similar to known fungal denitrifiers. Despite uncertainty in the placement of fungal *nirK* reads on the reference phylogeny (Fig. S4), we detected many of the dominant fungal taxa carrying *nirK*, as observed previously (33–35). Nevertheless, PCR-based approaches specifically targeting *nirK* reveal a more diverse community of soil fungal denitrifiers (36) than we observed in the metagenomes, where the presence of fungal *nirK* was rare and the majority were similar to known fungal denitrifiers. Notably, the *nirK* sequences that were obtained via the amplicon sequencing of arable soil that was sampled from a long-term field experiment showed more basal placements of fungal *nirK* in the phylogeny than did those detected in the metagenomes (36), underlining that the metagenomic *nirK* sequences mainly capture the most dominant of the fungal denitrifiers due to the limited sequencing depth. Still, 18.6% of the placements were found at the most basal part of the fungal tree, which implies the presence of eukaryotic organisms with *nirK* that were not captured by our reference phylogeny. These placements could point to unknown fungal denitrifiers, nonfungal eukaryotes, such as some algae (21) which carry *nirK* but are not represented in the phylogeny, or sequences from highly conserved portions of the alignment that are therefore difficult to parse. Nonetheless, the metagenomes captured the dominant terrestrial fungal denitrifiers, thereby contributing to a better understanding of their diversity, distribution, and ecology across global soils.

The abundance of fungal *nirK* in specific biomes was, in general, not explained by the overall abundance of fungi. Correlations with soil factors indicate that fungal denitrifiers are most abundant under conditions that are generally favorable for denitrification (4), with high moisture as well as C and N availability. However, their abundance increased with decreasing pH. Low pH has been shown to stimulate fungal denitrification (19), and Xu et al. (34) found a significant pH effect on the abundance of fungal *nirK*. Nevertheless, our results indicate that fungal denitrifiers appear to thrive at a higher pH, compared to fungi in general, and that they may also be less tolerant to dry conditions than is the overall fungal community. Alternatively, increased moisture would indicate decreased soil aeration, which could promote fungal denitrifiers due to their capacity for facultative respiration under anoxic conditions (37). Other relationships between the proportion of fungal *nirK* in the fungal community and soil properties displayed biome-specific patterns, likely because the total fungal *nirK* varies with the soil properties that distinguish the different biomes. For example, increasing inorganic N content generally promoted denitrifying fungi, relative to the overall fungal community, but negative associations to ammonium and nitrate were found in deserts and croplands, respectively. Because of losses by volatilization, desert soils often have lower ammonium levels than do forest and cropland soils (38). The increased ammonium levels in deprived desert soils have resulted in the depression of the fungal order Sordariales (39), and, indeed, a large fraction of the *nirK* sequence fragments that were found in the desert soil metagenomes were classified as Sordariales, supporting a negative relationship between the proportion of fungal denitrifiers within the fungal community and ammonium. Despite the positive correlation in forests, the negative association with soil nitrate and the proportion of denitrifying fungi in croplands aligns with the higher nitrate levels in croplands. This could indicate a nitrate threshold causing the restructuring of the fungal community. Similarly, plants might affect the proportion of fungal denitrifiers in the rhizosphere, as was observed for the lower abundance of fungal *nirK* with Poaceae. However, due to lack of data on the environmental conditions in the rhizosphere, it cannot be concluded whether these effects are driven by the host or the soil.

Fungal denitrifiers, relative to their prokaryotic counterparts, could be more important in tundra and forest soils, particularly boreal and temperate coniferous forests, compared to the other biomes where fungi are less prevalent, overall. The lower pH values in these systems, the negative correlation of the ratio between fungal and prokaryotic *nirK* fragment counts with pH, and the previous reports of fungal denitrification prevailing in acidic soils (18, 19) indicate that pH is a relevant predictor. In croplands, the increasing C/N is another possible driver, as is supported by Chen et al. (15), who reported that complex organic C substrates enhanced fungal denitrification, compared to that of bacteria. Nevertheless, even under the most favorable conditions, the abundance of fungal denitrifiers, relative to the prokaryotes, remains low and constitutes approximately 1% of all *nirK*-type denitrifiers. We based our counts of prokaryotic denitrifiers on *nirK* alone, as the other nitrate reductase among denitrifiers, namely, NirS, is much less abundant than is NirK in terrestrial ecosystems (40–43). Hence, adding the *nirS* counts would have a limited impact on the ratio between the fungal *nirK* and prokaryotic *nir* genes. Nevertheless, the low relative abundance of fungal *nirK* that was detected across the metagenomes indicates their potential contribution to denitrification, and the previously reported abundance, which was based on PCR-based approaches, may have been overestimated. The quantification of fungal *nirK* via quantitative PCR without sequence correction has recently been shown to be unreliable, as it overestimates the abundance of fungal denitrifiers by orders of magnitude (35, 36). Similarly, there is evidence that selective inhibition approaches that are commonly used to discriminate between fungal and prokaryotic denitrification overestimate the fungal contribution to denitrification (44). Overall, their roles in denitrification and their contributions to soil N_2_O emissions may therefore be less important than previously suggested.

Despite having a minor role in global denitrification, it remains intriguing why some fungi have the ability to denitrify. One explanation could be related to the saprotrophic and opportunistic pathogenic lifestyles that known denitrifying fungal taxa exhibit, which also involves the nitric oxide detoxification (45) that results from nitric oxide (NO) biosynthesis under the nitrosative stress that is caused by the host response during a fungal infection (46). Denitrification may also increase fungal virulence (31, 47) as discussed previously. For saprotrophs and opportunistic pathogens, metabolic flexibility and stress tolerance are advantageous, and denitrification further allows them to stay metabolically active in oxygen-depleted environments, such as host tissue. Our finding that many fungal denitrifiers are stress-tolerant cosmopolitans makes fungal denitrifiers potential beneficiaries of global climate change (48), with a potential for positive feedback through emissions of N_2_O. Moreover, warmer temperatures have been shown to increase soilborne fungal plant pathogens, and projections under different warming scenarios suggest an increase of these pathogens worldwide (49). Future work should aim at gaining a deeper understanding of the importance of this understudied fraction of the denitrifying microbial community in a global change context.

## MATERIALS AND METHODS

### Metagenome selection and biome assignment.

We searched the literature, National Center for Biotechnology Information (NCBI), and Integrated Microbial Genomes and Microbiomes (IMG/M hosted by the Joint Genome Institute) to construct a database of publicly available soil metagenomes that fulfilled the following criteria: (i) sequencing was done using Illumina short-read technology; (ii) a minimum of 100,000 reads of at least 150 nucleotides (nt); and (iii) the availability of metadata beyond geographic coordinates. The final database consisted of 1,980 metagenomes that represented 608 sampling locations around the globe (Fig. 1A).

The metagenomes were classified into three biome levels of increasing ecological complexity using the environment ontology (50), and, to discriminate between cropland and noncropland soils, the terrestrial biomes defined by Olson et al. (51) were used. Biome assignment was based on the GPS coordinates of each metagenome and was performed using the “sp” (52), “rgeos” (53), and “rgdal” (54) packages in R (4.1.2). The Level 1 biomes were categorized as terrestrial and host-associated, At Level 2, the biomes were distinguished into 6 terrestrial biomes, and these were further subdivided into 13 Level 3 biomes (Table S1). For those that were host-associated, we restricted our search to plants. Two of the Level 3 biomes (montane grasslands and shrublands, tropical and subtropical coniferous forests) were represented by just two and three samples, respectively, and were excluded from further analysis.

### Generation of *nirK* and 18S rRNA gene phylogenies.

We generated a *nirK* reference alignment and phylogeny to identify fungal, bacterial, and archaeal *nirK* sequences within the metagenomes. First, we updated and manually curated the alignment of the *nirK* sequences that were described in Graf et al. (55), which consisted of 3,450 sequences that were extracted from RefSeq genomes (NCBI), available on September 26, 2019. This alignment was converted into a hidden Markov model (HMM) and was used to search genome assemblies from NCBI GenBank using *hmmsearch* within the HMMER (v3.2.1) software package (56). The Bacterial and archaeal assemblies for this search were downloaded on October 7, 2021, and the fungal assemblies for this search were downloaded on November 21, 2021. Candidate *nirK* amino acid sequences were dereplicated at 100% identity using CD-HIT (v.4.8.1) (57) and were aligned against the original seed HMM. The alignments were evaluated and manually refined using a combination of ARB (v.6.1) (58) and FastTree (v.2.1.11) (59) to remove homologous sequences that lacked the conserved copper-binding motifs that were characteristic of the NirK protein (60) and to identify an appropriate multicopper oxidase outgroup that was detected by the HMM search. We also removed sequences originating from genomes that BUSCO (v5.3.1) (61) determined to be >5% contaminated and/or <90% complete, except for five >80% complete Omnitrophica metagenome-assembled genomes (MAGs), for which only medium quality assemblies were available. These bacterial sequences formed a sister clade to the fungi and were therefore critical in the discrimination of fungal versus nonfungal hits. Additional sequences were removed in cases in which the taxonomy that was entered by contributors in NCBI was in a different phylum than that which was determined by NCBI or in which the MMseqs2 taxonomy (v.fcf52600801a73e95fd74068e1bb1afb437d719d) (62) versus the UniRef50 database (63) indicated that *nirK*-containing contigs had interdomain contamination. Finally, we manually pruned the tree to remove tips with short terminal branch lengths that would have increased the computation time while not allowing better discrimination between the fungal and non-fungal gene fragment sequences.

The final *nirK* tree was generated with FastTree, using an amino acid alignment filter to exclude positions that were found in fewer than 5% of sequences and the poorly aligned terminal regions. It contains 6,732 sequences (including 373 outgroup sequences) and is comprised of 316 unique fungal *nirK* sequences (Fig. 2). The reference phylogeny for the fungal 18S rRNA gene was obtained using the reference sequences and tree from SILVA (SSU Ref NR 99 138.1 [64]). The tree was first manually pruned in ARB to retain only representative species of each of the major eukaryotic lineages. The RNA sequences were then transformed to DNA, prior to deduplication using BBMap (v.38.90) (65), which resulted in 3,559 sequences in the tree.

### Screening metagenomes for *nirK* and 18S rRNA gene fragments.

Fragments of *nirK* and 18S rRNA gene sequences were identified in metagenomes using GraftM (v. 0.13.1) (66). GraftM utilizes custom gene reference packages to search metagenomes by using HMMER and following this with the phylogenetic placement of the identified *nirK* gene fragments into a reference tree. For each gene, the tree model parameters that were required to run GraftM were calculated using RaxML (v.1.1.0) (67), and the tree was rerooted using FigTree (v.1.4.4) (68). We used only forward reads and ran GraftM with the default parameters, except for restricting the read length to 150, resulting in us screening only the first 150 nt of each read, regardless of the total read length. Our *nirK* identification method was validated by generating a mock data set of 24,670 150 nt fragments of the *nirK* sequences that were present in the phylogeny. This was followed by GraftM searches, using the specified parameters. Sensitivity was calculated by determining the fraction of eukaryotic *nirK* fragment sequences that were placed outside the fungal clade on the tree (13,117 of 13,266 reads, 98.8%). Specificity was determined by using 1,427,775 fragments that were derived from 19,037 multicopper oxidase family sequences that were identified in a database of bacterial and archaeal MAGs (69). Only 0.05% (102 of 195,039) sequences that were placed in the tree were inappropriately placed within the fungal group.

### Phylogenetic and statistical analyses.

We used a combination of the phylogenetic placement visualization and analysis tools guppy (v.1.1.alpha19-0-g807f6f3) (70) and gappa (v.0.8.0) (71) to remove nontarget *nirK* and 18S rRNA gene fragments and to classify *nirK* as being of bacterial, archaeal, or fungal origin. Since GraftM classifies reads based on their placement in the phylogenetic tree, we kept the most likely placement of each *nirK* fragment (i.e., the point mass) and counted it as belonging to the target microbial group if the mass of possible placements for a fragment sequence reached a threshold of one for the selected clade. The probability distribution of the most likely placements of each biome were visualized using the gappa examine “lwr” function and indicated the probability of the most likely placement, given as a likelihood weight ratio (Fig. S4). The placements within trees were visualized using iTOL v5 (72). To compare the aggregation of placements within fungal clades or for species among biomes, the placements were visualized for each biome individually in iTOL. Then, the trees were visually inspected for clades or leaves with placements that were mostly restricted to a single biome and with multiple placements.

Because of the inherent problem with the undersampling of functional genes in metagenomes having only few or zero gene fragment counts per metagenome (73), we excluded zero-valued samples and retained the 1,485 metagenomes that included both fungal *nirK* and fungal 18S rRNA gene fragment counts when reporting counts and comparing gene ratios across biomes. This minimizes zero-inflation within the data set and the subsequent skewing of the data, but it bears the risk of overrepresenting metagenomes with (multiple) gene fragment counts, which would result in inflated mean counts of functional genes.

All of the statistical analyses were carried out in the R environment (v.4.1.2). To account for the differences in the sequencing depth, the fungal *nirK* abundance was normalized by dividing the number of *nirK* fragment counts by the total number of reads in the corresponding metagenome. The fungal *nirK* fragment counts were also divided by the number of fungal 18S rRNA gene sequences that were detected so as to account for the differences in the total fungal abundance across samples. To compare the fungal *nirK* abundance with its prokaryotic counterparts, the fungal/prokaryotic *nirK* ratio was calculated. The effect of biomes on gene abundances was evaluated after the negative log-normal transformation of the data. A generalized linear model approach was chosen, utilizing a gamma distribution that was combined with a log link function (f*nirK*, f*nirK*/p*nirK*) or a Gaussian distribution (f*nirK*/18S) that was followed by an analysis of variance (ANOVA), using the “stats” (v.4.0.5) package. Levene’s test was used to test for the homogeneity of variances using the “car” package (74), and the model was visually inspected. The results are reported in Table S3. Pairwise and multiple comparisons of biomes were carried out using the Dunn-Sidák correction method within the “emmeans” package (75).

For the analysis of the potential factors driving the abundances of fungal *nirK*, ratios with overall fungal abundance, prokaryotic *nirK*, and a minimum of 25 metagenomes per biome were used to preserve statistical power. Further, the metadata that were associated with the metagenomes that contained fungal *nirK* were reduced to uncorrelated soil factors that were known to be relevant for denitrification (Table S2). These include different variables that were related to the soil C and N content, soil texture and moisture known to affect oxygen levels, soil pH, and copper content, as NirK is a copper-dependent nitrite reductase (76, 77). Spearman’s correlations were used to test for relationships between soil physicochemical variables and gene abundances or ratios, using the packages “corrr” (78) and “Hmisc” (79). Rhizosphere samples were excluded from this analysis, as metadata were largely missing. The fungal *nirK* abundance was instead compared across host plants.

### Data availability.

The metagenomes are available via the original publications or can be accessed by their NCBI BioProject numbers, as listed in Table S1. The *nirK* reference phylogeny, the genome accession numbers, and a table containing all of the extracted gene counts and metadata associated with the 1,980 metagenomes that were analyzed are deposited in Zenodo (doi:10.5281/zenodo.7292953).
